# Combined Metabolomic and Quantitative RT-PCR Analyses Revealed the Synthetic Differences of 2-Acetyl-1-pyrroline in Aromatic and Non-Aromatic Vegetable Soybeans

**DOI:** 10.3390/ijms232314529

**Published:** 2022-11-22

**Authors:** Man Zhao, Linlin Qian, Zhuoyu Chi, Xiaoli Jia, Fengjie Qi, Fengjie Yuan, Zhiqiang Liu, Yuguo Zheng

**Affiliations:** 1 Key Laboratory of Bioorganic Synthesis of Zhejiang Province, College of Biotechnology and Bioengineering, Zhejiang University of Technology, Hangzhou 310014, China; 2 The National and Local Joint Engineering Research Center for Biomanufacturing of Chiral Chemicals, College of Biotechnology and Bioengineering, Zhejiang University of Technology, Hangzhou 310014, China; 3 Hangzhou Sub-Center of National Soybean Improvement, Institute of Crop and Nuclear Technology Utilization, Zhejiang Academy of Agricultural Sciences, Hangzhou 310021, China

**Keywords:** aroma, vegetable soybean, 2-acetyl-1-pyrroline (2AP), metabolome, expression level, differential accumulation metabolites (DAMs)

## Abstract

Aroma is an important economic trait of vegetable soybeans, which greatly influences their market value. The 2-acetyl-1-pyrroline (2AP) is considered as an important substance affecting the aroma of plants. Although the 2AP synthesis pathway has been resolved, the differences of the 2AP synthesis in the aromatic and non-aromatic vegetable soybeans are unknown. In this study, a broad targeted metabolome analysis including measurement of metabolites levels and gene expression levels was performed to reveal pathways of aroma formation in the two developmental stages of vegetable soybean grains [35 (S5) and 40 (S6) days after anthesis] of the ‘Zhexian No. 8’ (ZX8, non-aromatic) and ZK1754 (aromatic). The results showed that the differentially accumulated metabolites (DAMs) of the two varieties can be classified into nine main categories including flavonoids, lipids, amino acids and derivatives, saccharides and alcohols, organic acids, nucleotides and derivatives, phenolic acids, alkaloids and vitamin, which mainly contributed to their phenotypic differences. Furthermore, in combination with the 2AP synthesis pathway, the differences of amino acids and derivatives were mainly involved in the 2AP synthesis. Furthermore, 2AP precursors’ analysis revealed that the accumulation of 2AP mainly occurred from 1-pyrroline-5-carboxylate (P5C), not 4-aminobutyraldehyde (GABald). The quantitative RT-PCR showed that the associated synthetic genes were 1-pyrroline-5-carboxylate dehydrogenase (*P5CDH*), ∆^1^-pyrroline-5-carboxylate synthetase (*P5CS*), proline dehydrogenase (*PRODH*) and pyrroline-5-carboxylate reductase (*P5CR*), which further verified the synthetic pathway of 2AP. Furthermore, the betaine aldehyde dehydrogenase 2 (*GmBADH2*) mutant was not only vital for the occurrence of 2AP, but also for the synthesis of 4-aminobutyric acid (GABA) in vegetable soybean. Therefore, the differences of 2AP accumulation in aromatic and non-aromatic vegetable soybeans have been revealed, and it also provides an important theoretical basis for aromatic vegetable soybean breeding.

## 1. Introduction

Soybean (*Glycine max* L.) is an important economic crop for providing vegetable oil and proteins to the world [1,2]. In recent years, the vegetable soybeans have become popular for their rich content of nutrients such as carbohydrates, proteins, vitamins, minerals, and so on. Moreover, aroma is also an important trait in vegetable soybeans, which influences their market price; hence, breeders started focusing on the aroma trait [3]. It has been found that the fragrance in soybeans is mainly due to the occurrence of 2-acetyl-1-pyrroline (2AP) [4,5]; 2AP is a principal aroma and flavor compound and widely exists in crops, vegetables, bacteria, fungi, animals and food products (for review see [6]). 

The compound 2AP is synthesized directly from methylglyoxal (MG) and 1-pyrroline (1-PYR) through a non-enzymatic pathway in which the hydrogen at position 2 is replaced by an acetyl group with a methyl ketone group [7]. Due to the pyrroline ring, 2AP is highly unstable, and its content in different food commodities and organisms varies greatly, ranging from 4 to 700 μg/kg [8,9,10]. Moreover, the content of 2AP also varies in different organs and developmental stages. For example, in aromatic soybeans, 2AP content was highest at the vegetative stage and then decreased gradually [11]. 

The synthetic pathway of 2AP and the main regulating factors have been revealed in different crops. In rice, it was found that γ-aminobutyraldehyde (GABald) is an important factor regulating the synthetic rate of 2AP through influencing the synthesis of the direct precursor of 2AP [12]. The betaine aldehyde dehydrogenase 2 (BADH2) has been identified as a major effector for rice aroma [13,14,15]. The results showed that in the presence of the functional *BADH2*, GABald is converted to 4-aminobutyric acid (GABA) inhibiting the synthesis of 2AP in fragrant rice, while the non-functional *BADH2* gene leads to the formation of 2AP [12]. Specifically, studies have reported that three SNPs and the deletion of eight bases in exon seven, and a seven-bases deletion in exon two of the *OsBADH2* gene leads to the formation of a premature termination codon and a non-functional *OsBADH2*, which is the main reason for the aroma of rice [15,16,17]. Further, mutants of the *OsBADH2* gene generated through RNAi or CRISPR/Cas9 have also conferred aroma to rice [18,19,20,21]. In addition, the mutations in *OsBADH1* also give rise to aroma in rice [14,22]. Similarly, in aromatic soybeans, a 2-base (TT) deletion in exon ten of *GmBADH2* (Glyma05g01770) results in a premature stop codon, leading to non-functional *GmBADH2* [23,24]. Suppression of the expression of the *GmBADH2* gene by RNAi or CRISPR-Cas9 also confers aroma to non-aromatic soybean [4,24]. Moreover, the synthesis of 2AP might also be affected by the expression of the *GAPDH* (Glyceraldehyde-3-phosphate dehydrogenase), *TPI* (Triosephosphate isomerase), *P5CR* (Pyrroline-5-carboxylate reductase), *PRODH* (Proline dehydrogenase), *P5CS* (Δ^1^-pyrroline-5-carboxylate synthetase), and *OAT* (Ornithine aminotransferase). The low expression level of *GAPDH* and the high expression levels of *TPI*, *P5CR*, *PRODH*, and *P5CS* in aromatic rice were found to be related to the synthesis of 2AP [9,25,26,27]. Similar expression patterns were also observed for *GAPDH* and *TPI* in aromatic soybeans, but the expression levels of *PRODH*, *P5CS* and *OAT* did not differ between aromatic and non-aromatic soybeans [5].

In recent years, targeted metabolomics based on the technology of UHPLC triple-quadrupole mass spectrometry (UHPLC_QQQ_MS) providing high throughput, rapid separation, high sensitivity and specificity, and wide coverage has become an effective method to investigate the diversity of metabolites in crops and vegetable species. For example, metabolomics analyses of soybean seeds and leaves revealed a large number of primary and secondary metabolites [28]. Many of these metabolites have been proved to have beneficial effects in their cardiac protective, anti-inflammatory, anti-cancer, anti-oxidant, or anti-obesity functions [29,30,31].

Due to the significant taste differences between the varieties of ZX8 and ZK1754, they were comprehensively compared to explore their diversity, differences and related reasons in metabolites. In particular, the differences of metabolites in the 2AP synthesis pathway between the two varieties were explored. In parallel, the expression levels of genes in the 2AP synthesis pathway were investigated. By combining the results of metabolomics and expression analysis, the major genes involved in the aroma trait development in the vegetable soybeans were revealed. This will provide a valuable basis for further improvements of vegetable soybeans.

## 2. Results

### 2.1. Metabolic Profiling of ZX8 and ZK1754 Grain at Two Developmental Stages

**Figure 1:**
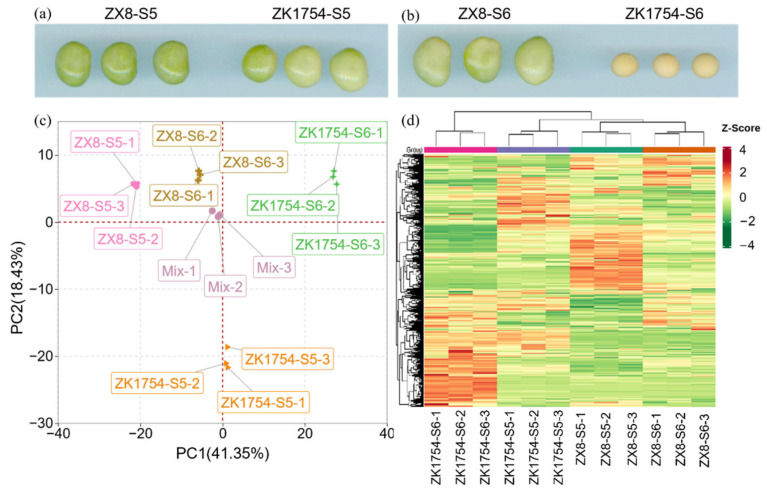
Metabolic changes in soybean grain at different developmental stages of ZX8 and ZK1754. (**a**,**b**) Photographs of grains at two stages of ZX8 (ZX8-S5, ZX8-S6) and of ZK1754 (ZK1754-S5, ZK1754-S6). (**c**) Metabolite principal component analysis (PCA), ‘mix’ was the pooled samples of each group, and three biological replicates were used for metabolite analysis. (**d**) Clustered heatmap of all metabolites. Each sample is represented by a column, each metabolite is represented by a row, the content of each metabolite is represented by a bar with a specific color, and the up- and down-regulated metabolites are represented by different degrees of red and green, respectively, with three biological replicates for metabolite analysis.

An extensive metabolic profiling was performed with grains of the varieties ZX8 and ZK1754 at two developmental stages, S5 and S6 (Figure 1a,b). A total of 636 metabolites were identified, which are mainly classified into nine categories, including flavonoids (15.72%), amino acids and derivatives (13.99%), lipids (12.74%), saccharides and alcohols (11.32%), phenolic acids (11.32%), organic acids (10.22%), nucleotides and derivatives (7.86%), terpenes (5.5%) and alkaloids (5.19%) (Table S1). The flavonoids represented the most abundant group with 100 flavonoids identified, and can be classified further into 25 flavonoids, 24 isoflavones, 19 flavonols, 14 dihydroflavones, nine flavone carboglycosides, five chalcones, two dihydroflavonols, one orange ketone and one flavanol (Table S1).

The PCA results indicated that the metabolites were significantly different between samples and developmental stages (Figure 1c). The percentage of the extracted two principal components (PC1 and PC2) were 41.35% and 18.43%, respectively, which could completely separate different vegetable soybeans and developmental stages. The result also showed that three biological replicates of each sample clustered closely, indicating that the test was reproducible and reliable. The heatmap (Figure 1d) confirmed that the metabolites accumulated in different developmental stages of the two soybean cultivars were significantly different. 

### 2.2. Differential Accumulation Metabolites (DAMs) in ZX8 and ZK1754 Soybean Grains 

To identify the significant DAMs, the analysis was executed based on the variable importance in projection (VIP) ≥ 1 and log_2_ fold change ≥ 2 and ≤0.5. Comparing the two soybean varieties, 171 DAMs (86 up-regulated, 85 down-regulated) were detected at stage S5 while 231 DAMs (128 up-regulated, 103 down-regulated) were detected at stage S6 (Table 1, Tables S2 and S3, Figure S1). The number of DAMs at stage S6 was clearly higher than at stage S5, but the up-regulated and the down-regulated ones were basically the same at both stages. Furthermore, the types of DAMs (as described above) were also consistent between the two stages. However, there was a clear difference in the coverage of different categories with flavonoids being most numerous at stage S5 (24.56%), while lipids represented the largest group at S6 (19.91%) (Figure S1, Tables S2 and S3). 

Comparing the two developmental stages with each other, 125 DAMs (40 up-regulated, 85 down-regulated) were detected between ZX8-S5 vs. ZX8-S6, and 196 DAMs (94 up-regulated, 102 down-regulated) were detected between ZK1754-S5 vs. ZK1754-S6 (Table 1, Tables S4 and S5). Notably, in both ZX8 and ZK1754, more down-regulated than up-regulated DAMs were observed at stage S6 than at stage S5 (Table 1). This was most significant in case of the free fatty acids with only one up-regulated and 38 down-regulated with log_2_ fold change > 2 and VIP > 1 (Table 1 and Table S4). On the other hand, in ZK1754 most of the amino acids and derivatives were down-regulated (19 down vs. 12 up) with the log_2_ fold changes > 2.2 (VIP > 1), yet most of free fatty acids (34 up-regulated, 14 down-regulated) were up-regulated with the log_2_ fold changes > 2.1 (VIP > 1) (Table 1 and Table S5). 

The results show significant differences in the content of metabolites between the aromatic (ZK1754) and non-aromatic (ZX8).

### 2.3. Special DAMs in Aromatic and Non-Aromatic Soybeans

**Figure 2:**
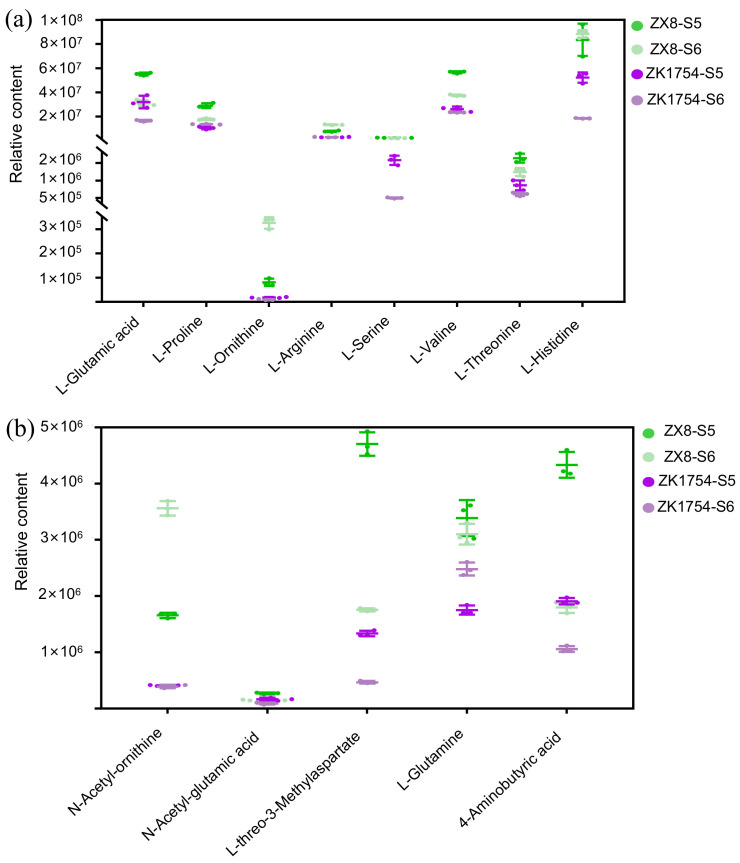
The content comparison of amino acids in ZX8 and ZK1754. (**a**) Plots of relative contents of amino acids accumulated at relatively high levels in ZX8 grain with three biological replicates for metabolite analysis. (**b**) Plots of relative contents of amino acid derivatives accumulated at relatively high levels in ZX8 grain with three biological replicates for metabolite analysis.

**Figure 3:**
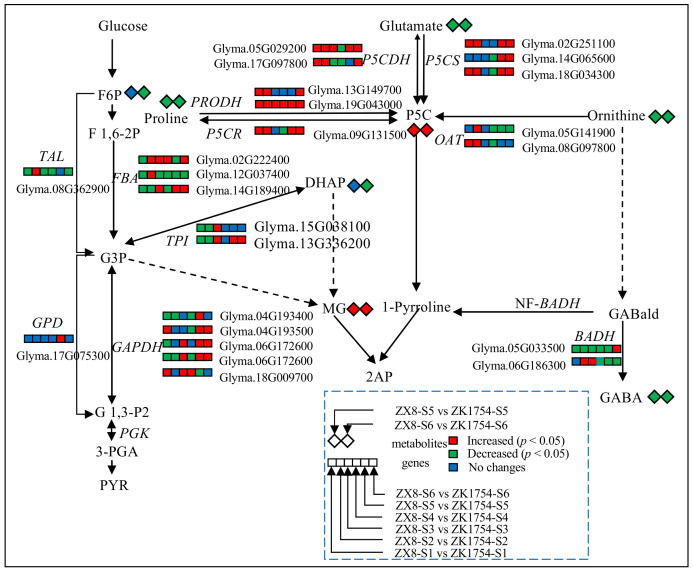
Specific synthesis pathway of 2AP in soybean. Genes or enzymes and substrates or products are shown in italics and normal, respectively. TAL: Transaldolase; FBA: Fructose-bisphosphate aldolase; TPI: Triosephosphate isomerase; P5CR: Pyrroline-5-carboxylate reductase; GPD: Glyceraldehyde-3-phosphate dehydrogenase NADP^+^; GAPDH: Glyceraldehyde-3-phosphate dehydrogenase; PRODH: Proline dehydrogenase; P5CDH: 1-Pyrroline-5-carboxylate dehydrogenase; P5CS: ∆^1^-Pyrroline-5-carboxylate synthetase; OAT: Ornithine aminotransferase; NF-BADH: Non-functional betaine-aldehyde dehydrogenase; BADH: Betaine-aldehyde dehydrogenase. F6P: Fructose 6-phosphate; F-1,6-P2: beta-D-Fructose-1,6-bisphosphate; G3P: Glyceraldehyde 3-phosphate; G 1,3-P2: Glycerate 1,3-diphosphate; 3-PGA: 3-Phosphoglycerate; PYR: Pyruvate; DHAP: Dihydroxyacetone phosphate; MG: Methylglyoxal; P5C: 1-Pyrroline-5-carboxylate; GABald: 4-Aminobutyraldehyde; GABA: 4-Aminobutyric acid.

According to a previous study [32], the DAMs can be roughly classified into two groups, the first of which is related to anti-oxidation and comprises flavonoids, phenolic acids, alkaloids and vitamins. The second group affects the taste and includes amino acids and derivatives, organic acids, as well as saccharides and alcohols. In our study, the concentration of antioxidant components was generally higher in ZK1754 than in ZX8 (Table 1, Tables S2, S3, S6 and S7). Metabolites related to taste did not show a common pattern, but individual components varied differently. For example, the concentrations of amino acids and derivatives in ZK1754 were lower than those in ZX8, while the organic acids, saccharides and alcohols varied differently between ZX8 and ZK1754 (Table 1, Tables S2, S3, S6 and S7). Specifically, the amino acids and their derivatives, such as L-glutamic acid, L-proline, L-ornithine, L-arginine, N-acetyl-ornithine, N-acetyl-L-glutamic acid, L-threo-3-methylaspartate, L-glutamine and 4-aminobutyric acid, were significantly lower in ZK1754 than in ZX8 (Figure 2a,b, Tables S2, S3, S6 and S7). Interestingly, L-glutamic acid, L-proline, L-ornithine and L-arginine are precursors of 2AP (Figure 3).

### 2.4. Quantification of the 2AP Synthesis-Related Metabolites

**Figure 4:**
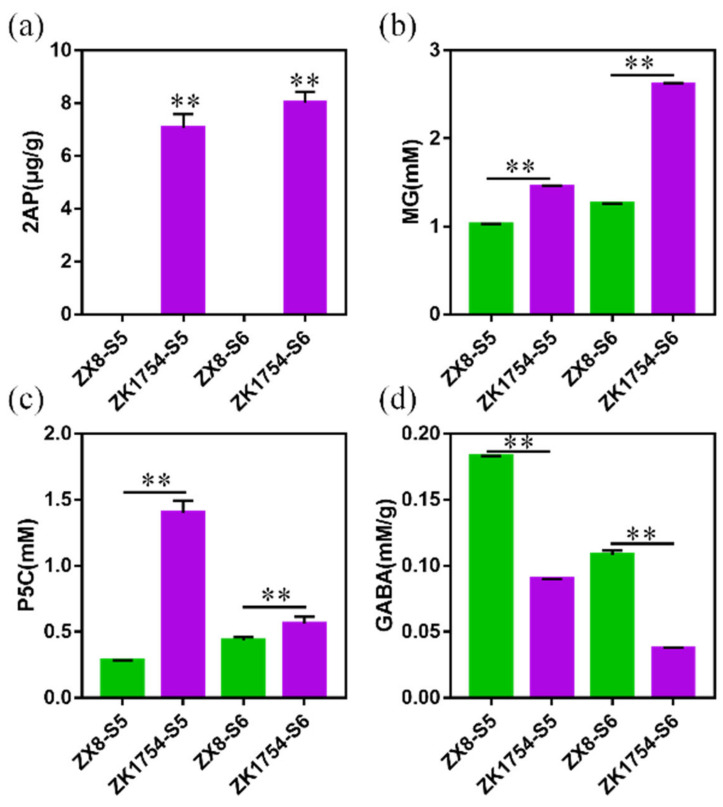
The content of 2AP and the main metabolites of its synthetic pathway. (**a**) 2AP content. (**b**) MG content. (**c**) P5C content. (**d**) GABA content. ** Indicates significance at the *p* < 0.01 level.

To further understand the differences in the 2AP synthesis in aromatic and non-aromatic soybean, the DAMs in the synthetic pathway of 2AP were compared in ZX8 and ZK1754. At first, the content of 2AP was measured and as expected 2AP could be found in ZK1754 at both stages with 7.21 µg/g and 8.09 µg/g, respectively. No 2AP content was detected in ZX8 (Figure 4a). As mentioned above, some of the 2AP-related metabolites, such as L-proline, L-glutamic acid, L-ornithine, L-arginine and GABA, were significantly down-regulated at ZK1754 (Figure 3, Table 2). Similarly, the metabolites in the amino acid branch pathway, 4-hydroxy-proline, 5-aminovaleric acid, N-acetyl-ornithine, N-acetyl-glutamate, L-threo-3-methylaspartate, α-ketoglutaric acid, succinic acid, guanidinoacetate, were also down-regulated (Table 2). In addition, the content of metabolites further away from 2AP in the synthetic pathway in the grains of ZK1754, such as F6P and DHAP in the glycolytic pathway, were also lower than that of ZX8 at S6 stage, but not at stage S5 (Figure 3, Table 2). 

Furthermore, compounds close to the 2AP in the synthetic pathway were quantified by chemical methods. The results showed that close precursors of 2AP, such as MG and P5C, were significantly higher in ZK1754 (*p* < 0.01) at both stages (Figure 4b,c). GABA, the competitive compound of 1-PYR, was also detected, and its contents in ZK1754 were significantly lower than in ZX8 (Figure 4d). Due to the instability of 1-PYR, its content was not detected.

The chemical assays were consistent with the metabolome data demonstrating the reliability of the metabolomics analyses (Figure 4d, Table 2). It remains to be elucidated how the different patterns of the amino acids and other 2AP precursors, and in particular their reduced contents in aromatic soybean, affect the biosynthesis of 2AP in ZK1754 and ZX8.

### 2.5. RT-PCR Analysis of the Genes in the 2AP Synthesis Pathway

**Figure 5:**
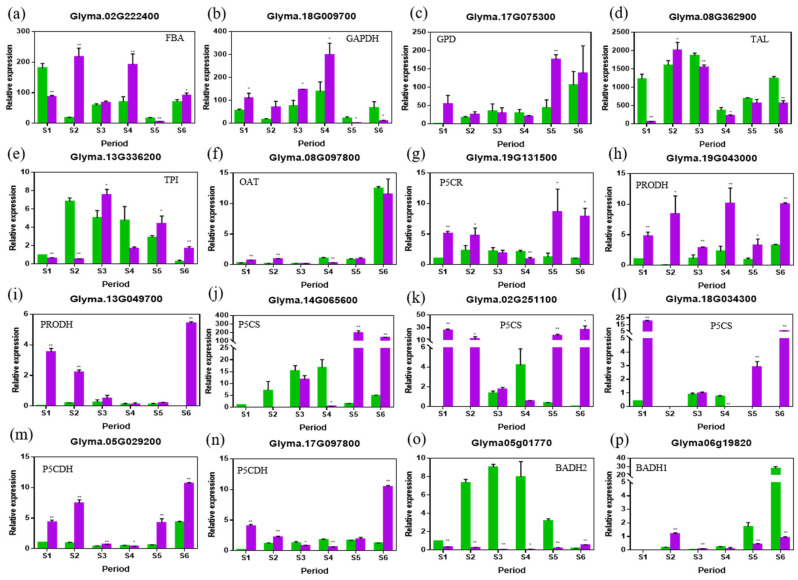
Relative expression levels of 2AP synthesis pathway-related genes in soybean grain (**a**–**p**). Green column: ZX8 (non-aromatic soybeans), violet column: ZK1754 (aromatic soybeans). S1, S2, S3, S4, S5, S6 for soybean grains correspond to 15, 20, 25, 30, 35, 40 days after anthesis. The actin gene was used as an internal reference. Experiments were performed using three independent biospecimens. Error bars: standard deviation. Significance was tested by comparing the expression of each gene in the green column at each period. * Indicates significance at the *p* < 0.05 level, ** indicates significance at the *p* < 0.01 level.

To reveal possible reasons for the differences in metabolite contents, eleven genes potentially involved in the 2AP synthetic pathway, *FBA*, *GAPDH*, *GPD*, *TAL*, *TPI*, *OAT*, *P5CR*, *PRODH*, *P5CS*, *P5CDH* and *BADH* were included in the expression analysis [9] (Figure 3). After combining the molecular evolution and expression analyses (Tables S8 and S9), the expression patterns of 16 candidate genes were analyzed at different developmental stages of seeds in ZK1754 and ZX8 (Table S10, Figure 5 and Figure S2). 

Generally, the expression patterns of these genes could be divided into three types. The type 1 expression varies at different stages of the soybeans and comprises the genes *FBA*, *GPD*, *TAL*, *TPI* and *OAT* (Figure 5). The type 2 also varies, but is mainly characterized by a higher expression in ZK1754, especially at the S5 and S6 stages, and comprises the genes *P5CR*, *PRODH*, *P5CS* and *P5CDH* (Figure 5). As can be concluded from the synthetic pathway of 2AP (Figure 3), these genes are likely to be vital for the synthesis of P5C from proline or glutamate. Therefore, their high expression levels in ZK1754 are consistent with the high content of 2AP. The type 3 expression comprises the three genes *BADH1*, *BADH2* and *GAPDH*, all of which are expressed at a lower level in ZK1754 compared to ZX8 at the S5 and S6 stages (Figure 5). As shown in Figure 3, BADH (BADH1 and BADH2) enzymes catalyze the synthesis of GABA from GABald. In ZX8, *BADH1* and *BADH2* were highly expressed at all stages of seed development (S1–S6). In ZK1754, only *BADH1* was basically expressed and the expression was low during the whole development. *GAPDH* was expressed higher in ZX8 than in ZK1754 at the S1–S4, but it was expressed oppositely at S5 and S6.

The results Indicated that the accumulation of the aromatic compound, 2AP, might be closely related to the expression levels of the type 2 and type 3 genes. The genes contributing to the synthesis of P5C were expressed at a significantly higher level in aromatic soybean seeds than in the non-aromatic ones. On the other hand, *BADH* genes involved in GABA and 2AP synthesis were expressed at a higher level in the non-aromatic soybeans.

## 3. Discussion

Aroma is an important trait of fresh soybean, which influences the market value. However, there are currently few studies on the metabolites and mechanisms related to the aroma development in fresh soybean. The main component contributing to the aroma of soybeans is 2AP. In this study, an extensive metabolic profiling of two developmental stages (S5 and S6) of two soybean cultivars (ZX8 and ZK1754) was performed. The expression of genes involved in the 2AP synthesis was also analyzed, and the results from both analyses will be discussed.

In general, the results revealed that the contents of important metabolites related to the 2AP synthesis, such as MG, P5C, 1-PYR, 2AP and GABA, were significantly different in aromatic and non-aromatic soybeans. Interestingly, the same trend was observed for the expression of genes involved in 2AP synthesis, such as *P5CR*, *PRODH*, *P5CS*, *P5CDH* and *BADH*.

Specifically, the contents of 2AP and its precursors, P5C and MG, were significantly higher in ZK1754 than in ZX8 (Figure 3 and Figure 4). This is in agreement with previous studies [5,7,9]. Furthermore, in our study, the expression levels of genes contributing to the synthesis of P5C, including *P5CDH*, *P5CS*, *PRODH* and *P5CR,* were higher in the aromatic soybean ZK1754 than in ZX8. Interestingly, although the *OAT* gene also contributes to the synthesis of P5C from ornithine, and *TPI* gene also contributes to the synthesis of MG, their expression trends were different from the other genes, and they were also different with the previous results [5,7,9,26]. The results indicated that the P5C might mainly come from the glutamate and proline pathways, not from the ornithine pathway. As for the pathway, obvious differences might have happened in different species. 

Interestingly, for the distant indirect substrates, such as proline, ornithine, glutamate and DHAP, it was found that their contents were significantly lower in ZK1754 than in ZX8. The reasons for this might include their participation in other metabolic pathways, such as the synthesis of proteins. In addition, GABald has also been reported to be able to transform into 1-PYR and GABA [33,34], and inhibit the synthesis of 2AP when the *BADH2* gene mutated [12]. In this study, there was a negative correlation between GABA and 2AP, but whether GABald inhibit the synthesis of 2AP needed to be further investigated. As for more distant compounds, such as G3P, DHAP, F6P and so on, their content and the expression of the related genes varied greatly, which could be influenced by many different metabolic pathways.

Previous studies found that the *BADH* genes are involved in the synthesis of 1-PYR and GABA [12,19,21,35]. In rice, two homologous genes, *BADH1* and *BADH2* with different degrees of the molecular divergence between different cultivars were detected [36,37]. The main gene contributing to the aroma of rice was found to be *BADH2* [12,37]. 

In our study, the expression patterns of *BADH1* and *BADH2* were different in the non-aromatic soybeans ZX8. *BADH1* was mainly expressed in the late stage (S6 stage) of seed development, while the *BADH2* expression gradually increased during seed development from stage S1 to S5 (Figure 5). However, in the aromatic soybean ZK1754, the expression of both *BADH1* and *BADH2* was generally low (Figure 5). Notably, the content of GABA was higher in ZX8 than in ZK1754 (Figure 3 and Figure 4) indicating that the accumulation of GABA was positively correlated with the *BADH* gene expression. 

As shown in previous studies [4,15,17,24,38], the *BADH2* gene can be mutated by the loss of two bases in the 10th exon, three SNPs and the deletion of eight bases in the seventh exon, or a seven-base deletion in the second exon. Our analysis of the *BADH2* gene sequence revealed that the similar mutation is present in ZK1754, in which there are two TT deletions in the tenth exon. 

Based on our findings, we conclude that two factors affect the accumulation of 2AP in aromatic soybean ZK1754. First, the synthesis of 1-PYR mainly originates from the synthesis pathway of P5C, not GABald. Second, the mutant *BADH2* gene controls the synthesis of 2AP. The specific mechanisms are unknown, and need to be investigated in the future. 

## 4. Materials and Methods

### 4.1. Plant Material

Soybean plants were grown naturally in the field, and their grains were taken every 5 days. S1, S2, S3, S4, S5, S6 for vegetable soybean grains correspond to 15, 20, 25, 30, 35, 40 days after anthesis (Figure S3). Sensory tests showed that aromatic vegetable soybean seeds had a strong aroma at later growth stages, so seeds at stages S5 and S6 were used for metabolic analysis. Seeds of non-aromatic soybeans (ZX8) and aromatic soybeans (ZK1754) were obtained from the Institute of Crop and Nuclear Technology Utilization, Zhejiang Academy of Agricultural Sciences.

### 4.2. Sample Extraction Procedure

Biological samples were freeze-dried in a vacuum freeze-dryer Scientz-100F (SCIENTZ, Ningbo, China). The freeze-dried samples were crushed using MM400 (RETSCH, Haan, Germany) with zirconia beads for 1.5 min at 30 Hz. Lyophilized powder (100 mg) was resuspended with 1.2 mL 70% methanol solution, vortexed for 30 s every 30 min for 6 times in total, then kept in a refrigerator at 4 °C overnight. Following centrifugation at 12,000 rpm for 10 min, the extracts were filtrated (SCAA-104, 0.22 μm pore size; ANPEL, Shanghai, China) before UPLC-MS/MS analysis.

### 4.3. Chromatography and Mass Spectrometry Acquisition Conditions

The sample extracts were analyzed using a UPLC-ESI-MS/MS system comprising a Nexera X2 UPLC (Shimadzu, Shanghai, China) and a QTRAP 4500 mass spectrometer (Sciex, Shanghai, China). An SB-C18 UPLC column (1.8 µm, 2.1 mm × 100 mm; Agilent, Shanghai, China) was used with a mobile phase consisting of solvent A (0.1% formic acid in water) and solvent B (0.1% formic acid in acetonitrile). A sample aliquot of 4 µL was injected, and the metabolites were separated with the following gradient program at a flow rate of 0.35 mL/min and an oven temperature of 40 °C: starting condition 5% B, linear increase to 95% B in 9 min, 95% B kept for 1 min, reduction of B to 5% in 0.1 min, 5% B kept for 3 min. The column outlet was connected to an ESI-triple quadrupole-linear ion trap (QTRAP)-MS.

The acquisition of the linear ion trap (LIT) and triple quadrupole (QQQ) scans were performed in positive and negative mode and controlled by the Analyst 1.6.3 software (AB Sciex) with the following parameters: turbo spray source temperature 550 °C; ion spray voltage (IS) 5500 V (positive ion mode)/−4500 V (negative ion mode); ion source gas I (GSI), gas II (GSII), curtain gas (CUR) were set at 50, 60, and 25.0 psi, respectively; the collision-activated dissociation (CAD) was high. Instrument tuning and mass calibration were performed with 10 and 100 μmol/L polypropylene glycol solutions in QQQ and LIT modes, respectively. QQQ scans were acquired as MRM experiments with collision gas (nitrogen) set to medium. DP and CE for individual MRM transitions was conducted with further DP and CE optimization. A specific set of MRM transitions was monitored for each period according to the metabolites eluted within this period.

### 4.4. Data Analysis

Unsupervised PCA (principal component analysis) was performed by statistics function prcomp within R (www.r-project.org (last accessed on 20 July 2022)). The data was unit variance scaled before unsupervised PCA. Significantly regulated metabolites between groups were determined by VIP ≥ 1 and absolute log_2_ FC (fold change) ≥ 1. VIP values were extracted from OPLS-DA result, which also contain score plots and permutation plots, generated using R package MetaboAnalyst R. The data was log transformed (log_2_) and centered (mean) before OPLS-DA. To avoid overfitting, a permutation test (200 permutations) was performed.

### 4.5. Content Determination

MG was determined by the o-phenylenediamine method with slight modifications. Soybean grain (0.3 g) was ground into powder in liquid nitrogen and added to 3 mL of 0.5 M perchloric acid, incubated in ice bath for 15 min, centrifuged for 10 min (4 °C, 12,000 rpm). The supernatant was transferred into a new centrifuge tube, the centrifugation repeated and 1.4 mL of supernatant pipetted into 600 μL of 7.2 mM o-phenylenediamine, and incubated for 20 min at room temperature. The absorbance value at 336 nm was measured and a standard curve for MG generated (Figure S4).

P5C content was determined according to a previous method with minor modifications [5,39]. Soybean grain (100 mg) was ground into powder in liquid nitrogen and added to 375 μL of 50 mM Tris-HCl (pH 8), 10% glycerol, 1% TritonX-100, 1% β-mercaptoethanol. The sample was rotated 5 times for 2 min in an ice bath, kept for 10 min at room temperature, centrifuged for 30 min at 4 °C, 14,000 rpm. The supernatant was transferred into a new centrifuge tube and 500 μL of 10% trichloroacetic acid and 125 μL of 40 μM O-aminobenzaldehyde were added. After standing for 30 min at room temperature and centrifuging for 10 min (4 °C, 14,000 rpm), the absorbance was measured at 440 nm. P5C concentration was calculated with A = εbc. A, absorbance; ε, extinction coefficient, 2.58 mM^−1^ cm^−1^; b, optical path length, 1 cm; c, solution concentration.

GABA content was determined using a previous method with slight modifications [40,41]. Then, 0.5 g of soybean grain was ground into powder in liquid nitrogen, 5 mL of 60% ethanol was added and the sample vortexed on a shaker for 4 h. After centrifugation for 3 min (4 °C, 8000 rpm), 0.6 mL of sodium tetraborate (0.2 M, pH 9) and 2 mL of 5% phenol/7% sodium hypochlorite were added to 1 mL of the supernatant. After heating at 100 °C for 5 min and cooling, the absorbance value was measured at 645 nm, and the GABA content was determined by a standard curve (Figure S5).

The contents of 2AP of aromatic and non-aromatic soybeans were measured by GC-IMS according to the following methods: fresh pods were harvested, and put on boiling water; after 5–10 min, the pods were dehulled and the grains were put into an incubator with GC-IMS. The detection conditions of the GC-IMS were as follows: analysis time 20 min, column type FS-SE-54-CB-1 15 m ID:0.53 mm, column temperature 60 °C, drift gas flow 150 mL/min, carrier gas/drift gas nitrogen, IMS temperature 45 °C, injection needle volume 400 μL, incubation time 15 min, incubation temperature 60 °C, injection needle temperature 65 °C. The concentration of 2AP was determined using an external standard.

### 4.6. RT-qPCR Analysis

The TOYOBO Kit (QPK-212) (TOYOBO, Shanghai, China) was used for RT-qPCR. The specific procedures were as follows: RNA was extracted from each sample and run on an agarose gel to ensure that the RNA was intact and of good purity. cDNA was synthesized from 1 μg RNA using a BioMarker Kit (No: RK02002) (BioMarker, Beijing, China). Exon-specific primers were used for expression level detection, and the product size was less than 300 bp (Table S11). RT-qPCR was performed in a total volume of 20 μL containing 10 μL SYBR^®®^ Green real-time PCR Master Mix -Plus- (2×), 2 μL Plus solution, 1.2 μL each of the primers (10 μM), 2.0 μL cDNA (diluted 6-fold), 3.6 μL ddH2O. Reaction program: pre-denaturation at 95 °C for 1 min, 55 cycles with denaturation at 95 °C for 10 s, annealing at 60 °C for 10 s, and extension at 72 °C for 20 s. Primer specificity was detected at 65–97 °C for 1 cycle with 3 biological replicates per sample.

## 5. Conclusions

In this study, comparative metabolomics and transcriptomes have been combined and carried out to reveal the aroma differences in the vegetable soybean varieties. The results suggested that there were nine different classes of DAMs in the ZK1754 and ZX8. The concentrations of amino acids involving in the synthesis of 2AP were lower in ZK1754 than in ZX8. The metabolites and RT-PCR analysis revealed that 2AP is mainly from P5C, not GABald. Moreover, the *GmBADH2* mutant was not only vital for the occurrence of 2AP, but also for the synthesis of GABA in vegetable soybeans. The results provide important significance for vegetable soybean breeding with aroma.

## Figures and Tables

**Table 1:** The classification and differential accumulation metabolites (DAMs) numbers in ZX8 vs. ZK1754.

Class	ZX8-S5 vs. ZK1754-S5		ZX8-S6 vs. ZK1754-S6		ZX8-S5 vs. ZX8-S6		ZK1754-S5 vs. ZK1754-S6	
	Up	Down	Up	Down	Up	Down	Up	Down
Flavonoids	37	5	33	9	3	6	9	10
Lipids	0	32	35	11	1	38	34	14
Amino acids and derivatives	4	21	4	29	10	6	12	19
Saccharides and Alcohols	17	2	8	13	14	3	8	16
Organic acids	7	7	7	11	3	6	6	11
Nucleotides and derivatives	8	5	19	10	2	12	14	12
Phenolic acids	6	4	11	9	1	9	5	8
Alkaloids	2	8	2	7	0	5	2	3
Vitamin	3	0	1	4	4	0	2	7
Others	1	1	8	0	1	0	2	0
Total	86	85	128	103	40	85	94	102

**Table 2:** Relative contents of amino acids and derivatives in ZX8 and ZK1754.

Metabolites	ZX8-S5	ZK1754-S5		ZX8-S6	ZK1754-S6		ZX8-S5	ZX8-S6		ZK1754-S5	ZK1754-S6	
	Relative Content			Relative Content			Relative Content			Relative Content		
L-glutamic acid	55,170,000	31,964,333	*	30,996,333	16,549,000	**	55,170,000	30,996,333	*	31,964,333	16,549,000	**
L-proline	29,087,667	10,533,167	**	17,831,333	13,705,000	**	29,087,667	17,831,333	**	10,533,167	13,705,000	**
L-ornithine	80,612	17,967	*	326,013	9045	**	80,612	326,013	**	17,967	9045	*
L-arginine	7,828,300	2,859,900	**	13,208,000	2,840,367	**	7,828,300	13,208,000	*	2,859,900	2,840,367	
γ-Aminobutyric acid	4,331,533	1,907,767	**	1,795,967	1,059,767	**	4,331,533	1,795,967	**	1,907,767	1,059,767	**
4-Hydroxy- proline	223,263	243,827		168,980	202,517		223,263	168,980	*	243,827	202,517	
5-Aminovaleric acid	217,463	194,113		183,020	156,257		217,463	183,020		194,113	156,257	
*N*-Acetyl-ornithine	1,656,333	410,120	**	3,561,533	388,720	**	1,656,333	3,561,533	**	410,120	388,720	
*N*-Acetyl-glutamic acid	268,810	165,237	*	149,453	89,232	**	268,810	149,453	*	165,237	89,232	**
L-threo-3-Methylaspartate	4,702,967	1,334,233	**	1,754,200	466,573	**	4,702,967	1,754,200	**	1,334,233	466,573	**
L-Glutamine	3,387,033	1,750,967	**	3,099,633	2,480,767	*	3,387,033	3,099,633		1,750,967	2,480,767	*
α-Ketoglutaric acid	381,923	320,593		384,210	276,077	*	381,923	384,210		320,593	276,077	
Isocitric Acid	9,293,833	9,384,800		10,395,433	11,808,667		9,293,833	10,395,433		9,384,800	11,808,667	
Citric Acid	31,133,667	32,903,333	*	36,366,667	34,530,333		9,293,833	36,366,667	**	32,903,333	34,530,333	*
Succinic Acid	43,160,333	39,516,000		29,892,000	7,596,200	**	43,160,333	29,892,000	*	39,516,000	7,596,200	**
Agmatine	45,548	33,690	*	44,307	49,773		45,548	44,307		33,690	49,773	*
Guanidinoacetate	9798	8694		10,489	7968		9798	10,489		8694	7968	
4-Guanidinobutanal	18,032,667	7,821,300	*	10,096,567	5,234,500	**	18,032,667	10,096,567	*	7,821,300	5,234,500	*
4-Guanidinobutyric acid	1,022,693	1,005,407		1,186,733	1,078,267		1,022,693	1,186,733		1,005,407	1,078,267	
4-Acetamidobutyric acid	34,808	27,498		82,552	69,309		34,808	82,552		27,498	69,309	
Dihydroxyacetone phosphate	76,280	110,893		122,330	9	**	76,280	122,330		110,893	9	**
D-Fructose 6-Phosphate	5,871,767	6,243,100		4,351,700	18,964	**	5,871,767	4,351,700		6,243,100	18,964	**

* Means *p* < 0.05, ** means *p* < 0.01.

## Data Availability

The datasets generated and/or analyzed during the current study are available from the corresponding author on reasonable request.

## Supplementary Materials

The following supporting information can be downloaded at: https://www.mdpi.com/article/10.3390/ijms232314529/s1.
