# Supplementary material for: Combined Metabolomic and Quantitative RT-PCR Analyses Revealed the Synthetic Differences of 2-Acetyl-1-pyrroline in Aromatic and Non-Aromatic Vegetable Soybeans
Source: Int J Mol Sci. 2022 Nov 22;23(23):14529. doi: 10.3390/ijms232314529 (PMC9738111; doi:10.3390/ijms232314529)

Figure S1. DAMs of ZX8 versus ZK1754. (a) ZX8-S5 vs. ZK1754-S5 differential metabolite heatmap. (b) ZX8-S6 vs. ZK1754-S6 differential metabolite heatmap. Each sample is represented by a column, each metabolite by a row, and the content of each metabolite is represented by a bar with a specific color, with up- and down-regulated metabolites represented by different degrees of red and green, respectively. Three biological replicates were used for metabolite profiling.

(a)

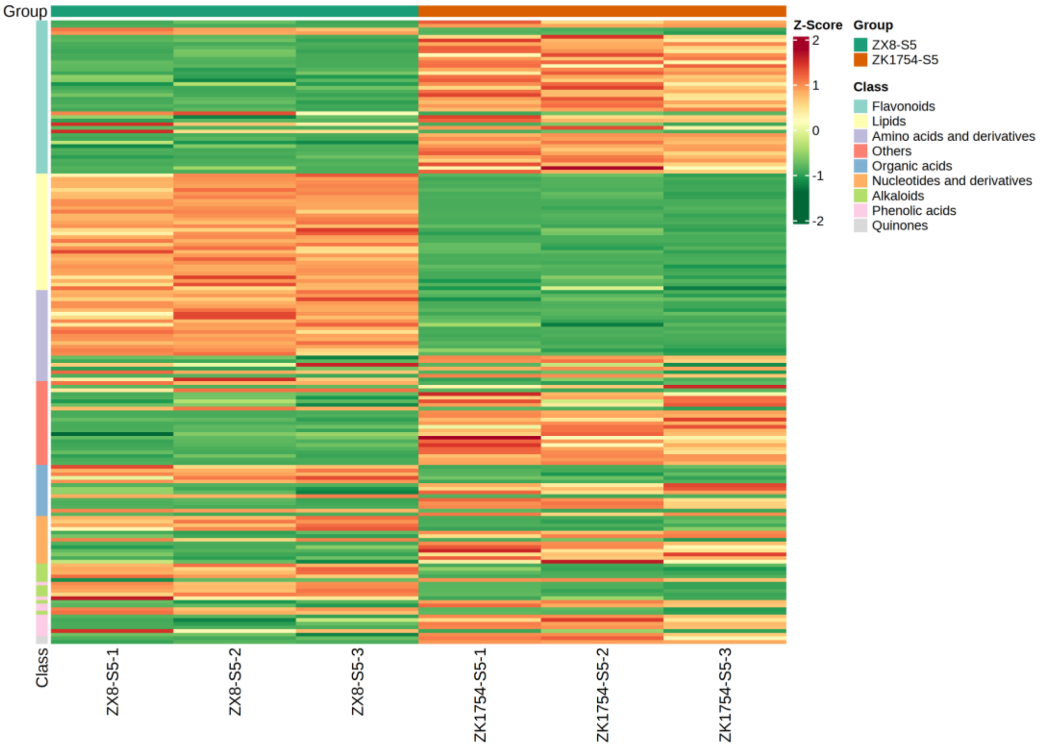

(b)

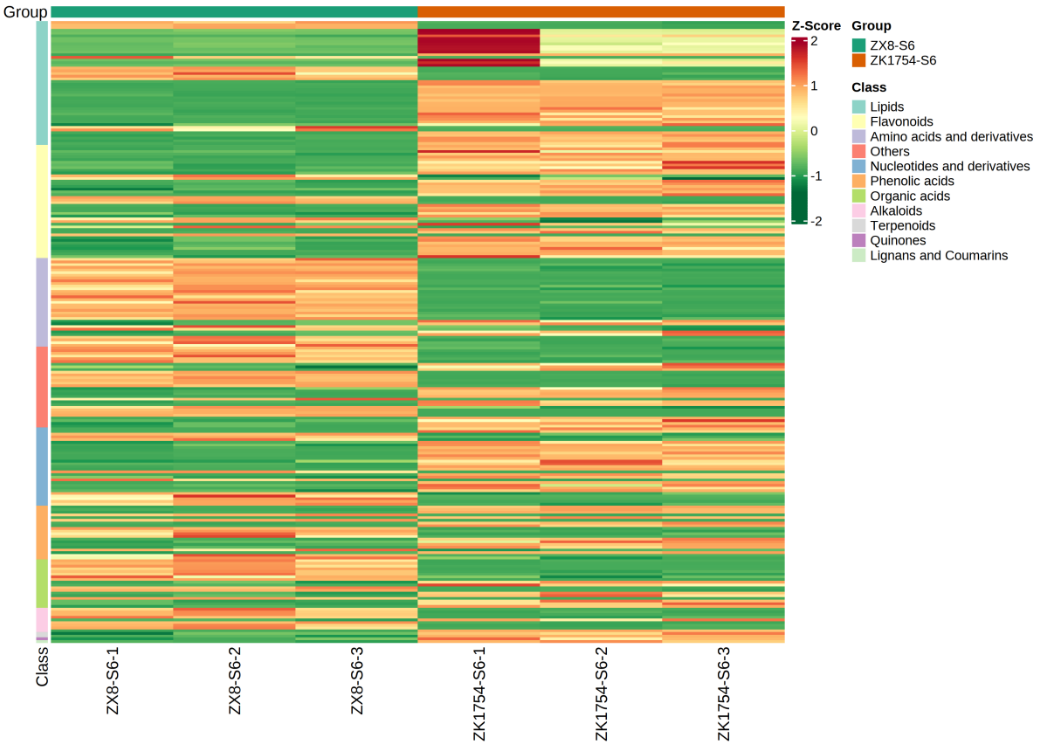

Supplement: Supplementary file 1 [file ijms-23-14529-s001.zip › ijms-1956011-supplementary/Figure S1.pdf]
