# Supplementary material for: Combined Metabolomic and Quantitative RT-PCR Analyses Revealed the Synthetic Differences of 2-Acetyl-1-pyrroline in Aromatic and Non-Aromatic Vegetable Soybeans
Source: Int J Mol Sci. 2022 Nov 22;23(23):14529. doi: 10.3390/ijms232314529 (PMC9738111; doi:10.3390/ijms232314529)

Figure S2. Relative expression levels of 2AP synthesis pathway related genes in soybean grain. Green column: ZX8 (non-aromatic soybeans), violet column: ZK1754 (aromatic soybeans). S1, S2, S3, S4, S5, S6 for soybean grains correspond to 15, 20, 25, 30, 35, 40 days after anthesis. * Indicates significance at the $p <$ 0.05 level, ** indicates significance at the $p < 0.01$ level.

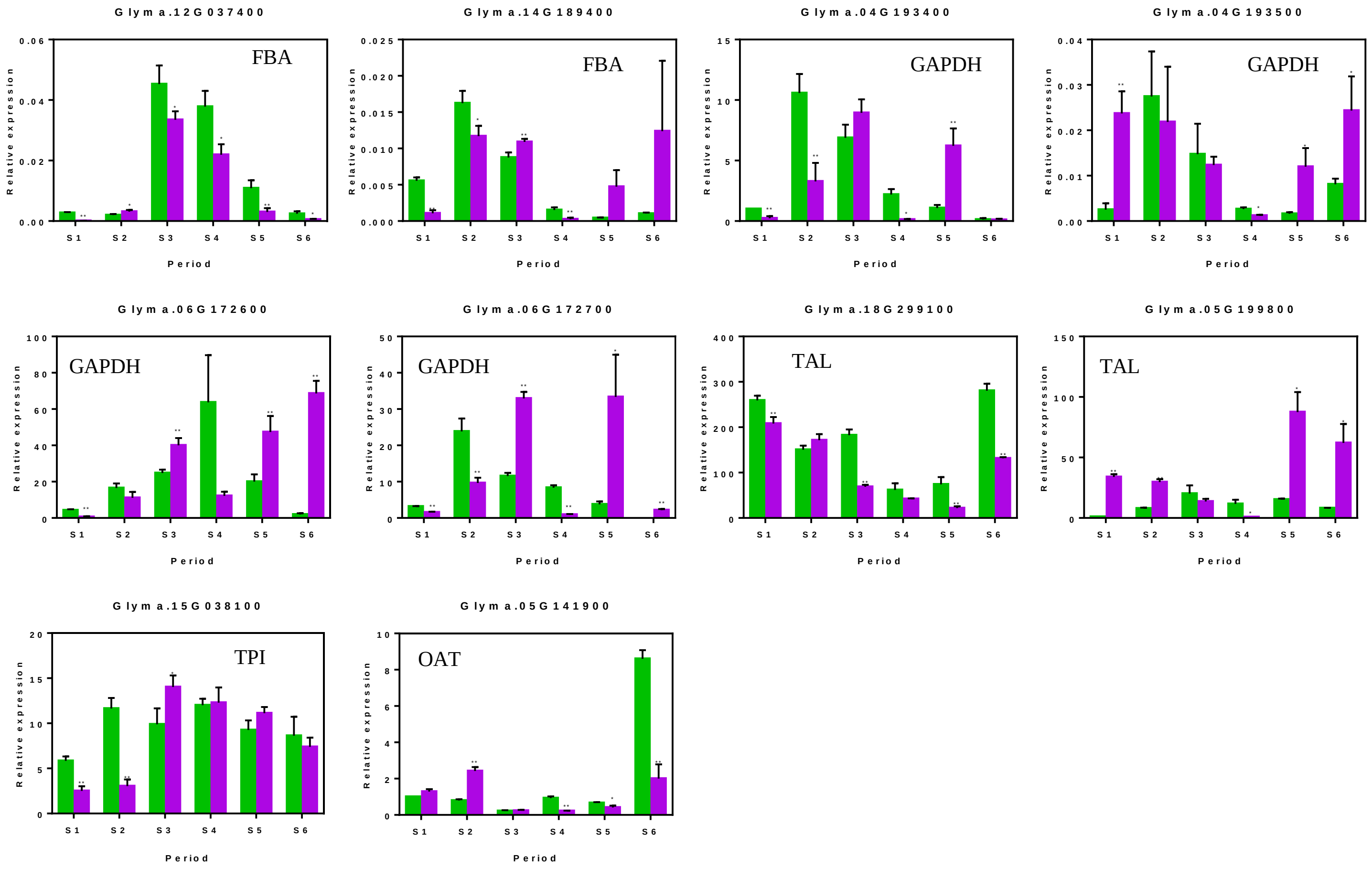

Supplement: Supplementary file 1 [file ijms-23-14529-s001.zip › ijms-1956011-supplementary/Figure S2.pdf]
