# Supplementary material for: Combined Metabolomic and Quantitative RT-PCR Analyses Revealed the Synthetic Differences of 2-Acetyl-1-pyrroline in Aromatic and Non-Aromatic Vegetable Soybeans
Source: Int J Mol Sci. 2022 Nov 22;23(23):14529. doi: 10.3390/ijms232314529 (PMC9738111; doi:10.3390/ijms232314529)
Supplement: Supplementary file 1 [file ijms-23-14529-s001.zip › ijms-1956011-supplementary/Table S10.pdf]

Table S10. Candidate genes and homologous genes in the 2AP synthesis pathway in soybean

Gene	Candidate gene				
FBA		Glyma.14G189400	Glyma.02G222400	Glyma.12G037400	
GAPDH	Glyma.06G172700	Glyma.04G193400	Glyma.06G172600	Glyma.04G193500	Glyma.18G009700
GPD		Glyma.17G075300	Glyma.03G027500		
TAL		Glyma.18G299100	Glyma.08G362900	Glyma.05G199800	
TPI		Glyma.15G038100	Glyma.13G336200		
OAT		Glyma.05G141900	Glyma.08G097800		
P5CR		Glyma.19G131500			
PRODH		Glyma.19G043000	Glyma.13G049700		
P5CS		Glyma.18G034300	Glyma.14G065600	Glyma.02G251100	
P5CDH		Glyma.05G029200	Glyma.17G097800		
BADH		Glyma05g01770	Glyma06g19820		
