# Supplementary material for: Combined Metabolomic and Quantitative RT-PCR Analyses Revealed the Synthetic Differences of 2-Acetyl-1-pyrroline in Aromatic and Non-Aromatic Vegetable Soybeans
Source: Int J Mol Sci. 2022 Nov 22;23(23):14529. doi: 10.3390/ijms232314529 (PMC9738111; doi:10.3390/ijms232314529)
Supplement: Supplementary file 1 [file ijms-23-14529-s001.zip › ijms-1956011-supplementary/Table S11.pdf]

Table S11. The primers used in this study

	Gene	Primer (5′ to3′)	Product size (bp)
β-Actin	Glyma.02G091900	F:GCATTGCAGATTGTAACCCTTT<br>R:CTCTCCTCACCTTCTGCAAATA	96
	Glyma.04G193400	F:CACGCTTTTCTCTACAGTCATG<br>R: GTAATGAAAGGGTCGTTAACGG	135
	Glyma.04G193500	F: CCTATTTACCTATCACTGCGGT<br>R: AGAAAGTGGTTGGAGTAGGAAG	97
GAPDH	Glyma.06G172600	F: GGAGAAAGAAGCTTCCTACGAT<br>R: TTGATCTGCTATCGCCGATAAA	131
	Glyma.06G172700	F: TAAATACGACAGTGTTCACGGA<br>R: GACTCAACAATGATGTCAGCTC	159
	Glyma.18G009700	F: GGTAGACGGCCTTATAGGTTTT<br>R: GACGTGGAAAACAAAATGTTGC	101
TPI	Glyma.13G336200	F: GAGGCAGCTTTTGAATGAATCA<br>R: CTGTTCAAGAGTTTCCCCAATG	106
	Glyma.15G038100	F: ATTGGAACAGGAAAGGTTGCTA<br>R: CTTACAGATGCAGCAACTTCAG	107
	Glyma.02G222400	F: TATTTGGTCAAACCTGTGCAAC<br>R: AAACACAAAGCCTCATTCACAG	88
FBA	Glyma.12G037400	F: AAAACACTGCCTTGAAGACATG<br>R: GTGTTGCTGCCTAACAAGTATC	213
	Glyma.14G189400	F: ACTCCTGGAAAGGGTATTCTTG<br>R: GAGATATTTAAGAGCACCGGGA	147
GPD	Glyma.17G075300	F: GCTGTTTCTGCTCTTCATATGG<br>R: GATGCATTGTAAGGAAGTCACC	115
	Glyma.05G199800	F: CAGTTTGCGGTGTTTGATAGAA<br>R: GTCATAGTAAGGGCTCTGTCTC	106
TAL	Glyma.08G362900	F: CGTCATGGTTAGTAATGCAGTG<br>R: AACGCACAACCATTGTATAGTG	199
	Glyma.18G299100	F: GGAAATTGTCCGCTTCAAGTAA<br>R: GCATGTGTTGTGTCATCGATTA	157
P5CDH	Glyma.19G131500	F: GCGCAAATCGAAATTGATTCTC<br>R: ATGAAGCCGAGAGTGTAGGATT	85
PRODH	Glyma.13G049700	F: GAATGTGGATGATGAAGTCGAG<br>R: GAGAAGAACGTCTCTTTCGTGG	85
	Glyma.19G043000	F: CACAAACACCCTTTGAGTTCAT<br>R: GTTGCCATTAAACTCTCGGAAG	101
	Glyma.02G251100	F: TAAAATAGCTGATGCCCTGGAA<br>R: GTTGGCAATAATTCGCATGTTG	169
P5CS	Glyma.14G065600	F: CCAGGCCATATCTTCAGAAGAA<br>R: TTTCATATCCTGCTTGTTGTGC	131
	Glyma.18G034300	F: TTGCTAAAAATAGCTGATGCCC<br>R: GTTCGTTTTAACACTCGACCAA	209

OAT	Glyma.05G141900	F: TGATCGTTGTGGTAGAGTCTTG<br>R: TATTGGAGCATAGTTTCCAGGG	137
	Glyma.08G097800	F: GTTGCAGAAGACAAAGCCAATA<br>R: CACATGCAATTCATACGTGTCT	109
P5CDH	Glyma.05G029200	F: ATCTTGCTGAGAGAAGAAAGCT<br>R: CAGCTTTGATCCTGGTATCTCA	116
	Glyma.17G097800	F: CAGGATCAAAGTTGCTATTCGG<br>R: TGACTGTTTTGATAGTCCGTGA	187
BADH	Glyma06g19820	F: TACATATCTGATGAACCGTGGG<br>R: GATAAAATCAACTCAGCCCGTC	200
	Glyma05g01770	F: TGAAGCGGGTGCTCCTTTAG<br>R: AATATGGTCCATTCAGCAGC	195
