# Supplementary material for: Combined Metabolomic and Quantitative RT-PCR Analyses Revealed the Synthetic Differences of 2-Acetyl-1-pyrroline in Aromatic and Non-Aromatic Vegetable Soybeans
Source: Int J Mol Sci. 2022 Nov 22;23(23):14529. doi: 10.3390/ijms232314529 (PMC9738111; doi:10.3390/ijms232314529)
Supplement: Supplementary file 1 [file ijms-23-14529-s001.zip › ijms-1956011-supplementary/Table S8.pdf]

Table S8. The genes related to the synthesis of 2AP and their homologs in plants

Gene	Species	Homologous gene
GAPDH	*Oryza sativa* L.	LOC_Os04g40950.1<br>LOC_Os02g38920.1(94.7%) LOC_Os08g03290.1(94.4%)<br>LOC_Os06g45590.1(82.8%) LOC_Os02g07490.1(82.5%)<br>LOC_Os04g38600.1(65.6%) LOC_Os03g03720.1(67.4%)
	Ananas comosus (Linn.) *Merr.*	Aco003511.1(81.3%) Aco018596.1(81.0%) Aco004676.1(78.9%)<br>Aco007469.1(64.7%) Aco011924.1(62.0%) Aco016173.1(24.6%)
	Brassica oleracea L.	Bol010398(93.5%) Bol031435(93.5%) Bol029171(93.2%)<br>Bol038208(82.5%) Bol021505(82.2%) Bol033485(68.2%)<br>Bol042826(65.3%) Bol031102(65.3%) Bol031393(65.6%)
	Glycine max (Linn.) *Merr.*	Glyma.06G172700.1(93.8%) Glyma.04G193400.1(93.8%)<br>Glyma.06G172600.1(93.8%) Glyma.04G193500.1(93.5%)<br>Glyma.18G009700.1(91.7%) Glyma.11G247600.1(91.7%)<br>Glyma.19G078300.1(92.6%) Glyma.05G073100.1(93.2%)<br>Glyma.03G092700.1(74.8%) Glyma.16G081400.1(75.1%)<br>Glyma.19G106800.1(56.4%) Glyma.16G044900.1(56.4%)<br>Glyma.06G015900.1(58.5%) Glyma.04G015900.1(58.5%)<br>Glyma.16G150200.1(35.0%)
TPI	*Oryza sativa* L.	LOC_Os01g62420.1<br>LOC_Os01g05490.1(89.4%) LOC_Os09g36450.1(70.6%)
	Ananas comosus (Linn.) *Merr.*	Aco015453.1(89.4%) Aco024987.1(84.3%) Aco013124.1(69.8%)<br>Aco020079.1(63.1%)
	Brassica oleracea L.	Bol044218(90.6%) Bol044217(89.0%) Bol045882(71.4%)<br>Bol006738(71.4%) Bol045407(54.5%) Bol045406(38.0%)
	Glycine max (Linn.) *Merr.*	Glyma.15G038100.1(85.5%) Glyma.13G146200.1(71.8%)<br>Glyma.13G336200.1(68.2%) Glyma.03G185800.1(67.5%)<br>Glyma.19G186000.1(66.7%) Glyma.10G059500.1(67.1%)
FBA	*Lycopersicon esculentum Miller*	Solyc09g009260<br>Solyc10g083570.1.1(96.4%) Solyc07g065900.2.1(87.7%)<br>Solyc05g008600.2.1(71.2%) Solyc02g062340.2.1(70.9%)<br>Solyc01g110360.2.1(69.8%) Solyc02g084440.2.1(70.4%)<br>Solyc10g054380.1.1(27.4%) Solyc10g054390.1.1(22.3%)<br>Solyc10g054340.1.1(9.8%)
	Oryza sativa L.	LOC_Os01g67860.1(91.3%) LOC_Os05g33380.1(90.8%)<br>LOC_Os10g08022.1(90.2%) LOC_Os08g02700.1(86.9%)<br>LOC_Os06g40640.1(86.3%) LOC_Os11g07020.1(70.7%)<br>LOC_Os01g02880.1(70.4%)
	Ananas comosus (Linn.) *Merr.*	Aco025753.1(91.9%) Aco024201.1(91.9%) Aco007775.1(88.5%)<br>Aco003226.1(71.8%) Aco021826.1(71.5%) Aco007583.1(13.4%)
	Brassica oleracea L.	Bol010037(92.2%) Bol037713(91.6%) Bol004435(90.8%)<br>Bol025079(90.2%) Bol039645(87.7%) Bol037714(84.9%)<br>Bol045872(70.1%) Bol014852(71.2%) Bol018526(68.7%)<br>Bol028791(69.3%) Bol016047(68.2%) Bol006726(67.9%)

	Glycine max (Linn.) *Merr.*	Glyma.14G189400.1(92.2%) Glyma.13G151800.1(91.9%) Glyma.02G222400.1(91.6%) Glyma.03G191500.1(91.6%) Glyma.10G066700.1(91.6%) Glyma.14G010900.1(88.5%) Glyma.02G303000.1(88.0%) Glyma.12G037400.1(71.2%) Glyma.11G111400.1(71.5%) Glyma.11G111100.1(71.5%) Glyma.04G008300.1(70.7%) Glyma.20G122500.1(70.7%) Glyma.10G268500.1(70.7%) Glyma.12G153000.1(68.4%)
TAL	*Arabidopsis thaliana* (L.) *Heynh.*	At5g13420
	Oryza sativa L.	LOC_Os01g70170.1(75.8%)
	Ananas comosus (Linn.) *Merr.*	Aco015068.1(79.7%)
	Brassica oleracea L.	Bol043436(95.0%) Bol034232(55.0%)
	Glycine max (Linn.) *Merr.*	Glyma.18G299100.1(86.8%) Glyma.08G362900.1(86.5%) Glyma.05G199800.1(66.9%) Glyma.08G277700.1(53.7%) Glyma.15G205300.1(53.7%) Glyma.02G189100.1(52.3%) Glyma.13G043400.1(52.7%) Glyma.03G072600.1(52.5%) Glyma.08G007300.1(35.6%) Glyma.18G139200.1(21.5%)
PRODH	*Oryza sativa* L.	LOC_Os10g40360.1
	Ananas comosus (Linn.) *Merr.*	Aco012041.1(58.2%)
	Brassica oleracea L.	Bol040145(62.0%) Bol033129(60.6%) Bol032297(61.2%) Bol043056(60.0%)
	Glycine max (Linn.) *Merr.*	Glyma.19G043000.1(56.9%) Glyma.13G049700.1(56.5%) Glyma.18G278900.1(56.3%) Glyma.19G042900.1(56.3%) Glyma.08G255900.1(51.0%)
P5CS	*Oryza sativa* L.	LOC_Os05g38150(P5CS1) LOC_Os01g62900(P5CS2) LOC_Os01g73450.1(7.7%)
	Ananas comosus (Linn.) *Merr.*	Aco015423.1(80.0%) Aco014596.1 1(8.3%)
	Brassica oleracea L.	Bol016209(87.4%) Bol044229(86.9%) Bol020440(86.6%) Bol044364(84.9%) Bol025367(84.1%)
	Glycine max (Linn.) *Merr.*	Glyma.18G034300.1(88.0%) Glyma.14G065600.1(87.3%) Glyma.02G251100.1(87.2%) Glyma.01G099800.1(86.2%) Glyma.03G069400.1(84.8%) Glyma.18G188000.1(80.3%) Glyma.07G137300.1(80.0%)
OAT	*Oryza sativa* L.	LOC_Os03g44150.1 LOC_Os05g03830.1(42.3%) LOC_Os04g52440.1(44.4%) LOC_Os08g10510.1(45.0%) LOC_Os04g52450.1(45.2%) LOC_Os02g02210.1(44.6%) LOC_Os05g39770.1(45.0%) LOC_Os03g21960.1(41.6%) LOC_Os03g07570.4(39.7%) LOC_Os07g27780.1(26.8%) LOC_Os08g41990.1(32.6%) LOC_Os08g14770.1(19.7%)
	Ananas comosus (Linn.) *Merr.*	Aco005104.1(83.3%) Aco001566.1(39.7%) Aco007099.1(44.0%) Aco018682.1(41.9%) Aco004116.1(36.8%)
	Brassica oleracea L.	Bol006334(80.8%) Bol032105(23.7%) Bol023248(46.3%)
	Glycine max (Linn.) *Merr.*	Glyma.05G141900.1(77.0%) Glyma.08G097800.1(76.5%) Glyma.08G245800.1(22.2%) Glyma.17G184700.1(22.8%) Glyma.19G189900.1(11.8%) Glyma.02G189300.1(9.1%) Glyma.15G181100.1(9.1%)
