# Supplementary figures and images for: Combined Metabolomic and Quantitative RT-PCR Analyses Revealed the Synthetic Differences of 2-Acetyl-1-pyrroline in Aromatic and Non-Aromatic Vegetable Soybeans
Source: Int J Mol Sci. 2022 Nov 22;23(23):14529. doi: 10.3390/ijms232314529 (PMC9738111; doi:10.3390/ijms232314529)

**Figure S3.** Seed photos of ZX8 and ZK1754 at different developmental stages

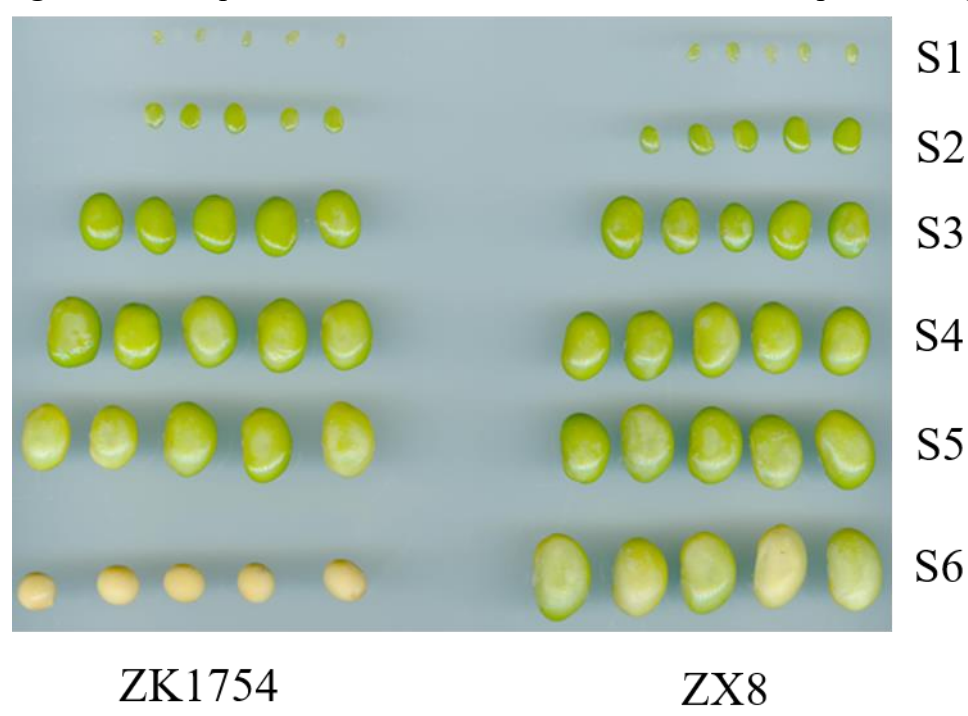

Supplement: Supplementary file 1 [file ijms-23-14529-s001.zip › ijms-1956011-supplementary/Figure S3.pdf]

**Figure S4.** Standard curve of MG

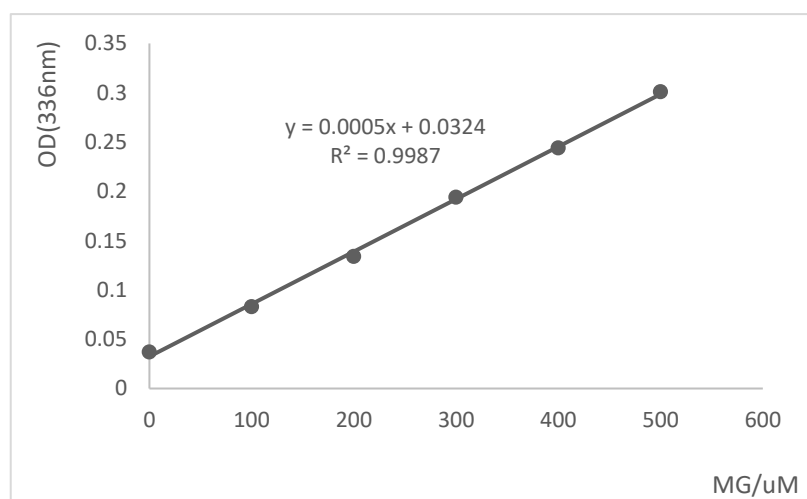

Supplement: Supplementary file 1 [file ijms-23-14529-s001.zip › ijms-1956011-supplementary/Figure S4.pdf]

**Figure S5.** Standard curve of GABA

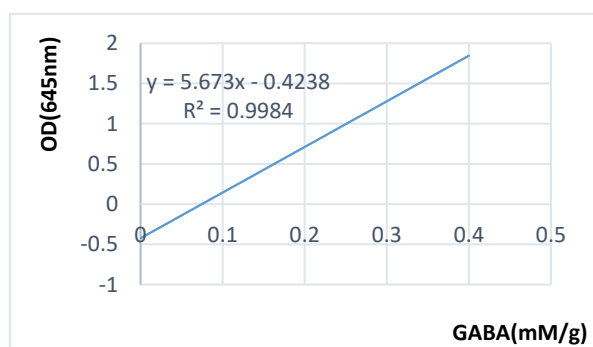

Supplement: Supplementary file 1 [file ijms-23-14529-s001.zip › ijms-1956011-supplementary/Figure S5.pdf]
